# Nut Allergy: Clinical and Allergological Features in Italian Children

**DOI:** 10.3390/nu13114076

**Published:** 2021-11-15

**Authors:** Sylvie Tagliati, Simona Barni, Mattia Giovannini, Giulia Liccioli, Lucrezia Sarti, Tatiana Alicandro, Erika Paladini, Giancarlo Perferi, Chiara Azzari, Elio Novembre, Francesca Mori

**Affiliations:** 1 Pediatric Unit, Sant’Anna University Hospital of Ferrara, 44124 Ferrara, Italy; sylvie.tagliati@edu.unife.it; 2 Allergy Unit, Department of Pediatrics, Meyer Children’s University Hospital, 50139 Florence, Italy; simonabarni@hotmail.com (S.B.); giulialiccioli@gmail.com (G.L.); lucrezia.sarti@unifi.it (L.S.); elio.novembre@unifi.it (E.N.); francesca.mori@meyer.it (F.M.); 3 Division of Allergy and Clinical Immunology, Dermatology Unit, Surgical, Medical and Dental Department of Morphological Science Related to Transplant, Oncology and Regenerative Medicine, University of Modena and Reggio Emilia, 41124 Modena, Italy; tatiana.alicandro@gmail.com; 4 Pediatric Respiratory and Allergy Unit, Women’s and Children’s Health Department, University of Padua, 35128 Padua, Italy; erikapaladini@yahoo.it; 5 Section of Pediatrics, Department of Health Sciences, University of Florence and Meyer Children’s Hospital, 50139 Florence, Italy; giancarlo.perferi@meyer.it (G.P.); chiara.azzari@unifi.it (C.A.)

**Keywords:** children, nut allergy, oral food challenge, peanut, prick by prick, serum specific IgE, skin prick test, tree nut

## Abstract

Background: Nut allergies are an increasingly frequent health issue in the pediatric population. Tree nuts (TN) and peanuts are the second cause of food anaphylaxis in Italy. Unfortunately, knowledge of the clinical characteristics of a TN allergy in Italian children is limited. Our study aimed to identify the clinical and allergological characteristics of Italian children with a nut allergy (TN and peanut). Methods: A retrospective observational analysis was performed on the clinical charts of children with a history of nut reaction referred to the allergy unit of the hospital from 2015 to 2019. The studied population was represented by children with a confirmed nut allergy based on positive prick by prick and/or serum-specific IgE to nut plus a positive nut oral food challenge. Demographic, clinical, and allergological features were studied and compared among different nuts. Results: In total, 318 clinical charts were reviewed. Nut allergy was confirmed in 113 patients. Most patients (85/113, 75%) had a familial history of allergy and/or a concomitant allergic disorder (77/113, 68%). Hazelnut and walnut were the more common culprit nuts observed in allergic children. Anaphylaxis was the first clinical manifestation of nut allergy in a high percentage of children (54/113, 48%). The mean age of the first nut reaction was statistically higher with pine nuts. Over 75% of children reported a single nut reaction. During the OFCs, the signs and symptoms involved mainly the gastrointestinal system (82/113, 73%) and resolved spontaneously in most cases. Severe reactions were not frequent (22/113, 19%). Conclusion: To our knowledge, this is the first Italian study that provided a comprehensive characterization of children with a nut allergy. These results are important for clinicians treating children with a nut allergy.

## 1. Background

Nut allergies are an emerging health issue in the pediatric population [1], which are experiencing increasing prevalence in childhood and exhibiting important effects on the quality of life of children and their families [2,3,4,5]. Tree nuts (TN) and peanuts have been identified as the main culprits of fatal or near-fatal anaphylaxis, even with consumption in a small amount [6,7]. In Italy, TN and peanuts are the second-leading cause of food anaphylaxis and the first in North America [8,9]. TN include almonds, Brazil nuts, cashews, hazelnuts, macadamia nuts, pecan nuts, pine nuts, pistachios, and walnuts. On the contrary, peanuts are not considered as TN because they belong to the *Fabaceae* family and are classified as legumes [10]. In this study, for convenience, we used the term nut, which includes TN and peanuts.

Clinically, a nut allergy can present as a primary nut allergy or pollen food syndrome (PFS)/oral allergic syndrome (OAS). The primary nut allergy is usually characterized by systemic and severe reactions due to the presence of serum-specific IgE (s-IgE) against the major nut storage proteins (e.g., Ara h 2 for peanuts). Instead, PFS/OAS is usually characterized by mild and isolated signs and symptoms to the oropharynx. PFS/OAS manifests in patients with seasonal allergic rhinitis and a history of reaction to nuts due to the presence of s-IgE directly against heat-labile proteins (e.g., PR-10), homologous to those in pollen [8,11].

The diagnosis of a nut allergy is based on clinical history, prick by prick (PbP) results, and s-IgE detection [12,13]. Molecular allergen analysis is becoming a more utilized method and may improve accuracy for diagnosing [13]. The oral food challenges (OFCs) are still considered the gold standard for the diagnosing of nut allergies and are useful to distinguish between sensitization and a primary allergy [14].

The knowledge of clinical characteristics of nut allergies in Italy is markedly limited, especially in the pediatric population [15]. Hence, our study aimed to identify the demographic, clinical, and allergological characteristics of Italian children with different nut allergies, comparing these features between the various nuts.

## 2. Materials and Methods

We performed a retrospective observational analysis of the clinical charts of children with a history of nut reactions who were referred to the allergy unit of the hospital from January 2015 to December 2019. Written informed consent for all performed procedures was obtained from the children’s parents. The code of the event report issued by the hospital is IR904-18-26854.

A skin prick test (SPT) for aeroallergens (including grass, artemisia, cypress, olive tree, hazel, birch, and poplar) and/or specific foods if the clinical history was suggestive of respiratory allergy (asthma and/or oculorhinitis) and food allergy was performed by using commercial extracts (Lofarma, Milan, Italy). When an SPT for specific foods was not available, we performed a PbP with fresh foods. Fresh nuts were used for PbPs, according to Ortolani et al. [16]. Both SPTs and PbPs were performed on the volar surface of the forearm with a lancet, as per the European standard [17]. The results were read after 15 min: a wheal diameter ≥ 3 mm was considered positive. Positive and negative controls were used—histamine (10 mg/mL; Lofarma, Milan, Italy) and normal saline, respectively.

In patients with a positive PbP to nuts, a s-IgE to nuts and the available molecular components (peanuts: (Ara h 1, Ara h 2, Ara h 3, Ara h 8, Ara h 9); hazelnuts: (Cor a 1, Cor a 8, Cor a 9, Cor a 14); walnuts: (Jug r 1, Jug r 3)) were detected by ImmunoCAP (Thermo Fisher Scientific, Uppsala, Sweden), following the manufacturer’s instructions. A positive cut-off point was set at 0.1 kUA/L.

All the patients with positive tests in reference to the culprit nut underwent an OFC with the nut suspected as the cause for the reaction or beginning with the nut suspected as the cause for the first reaction in chronological order in the case of multiple reactions (according to clinical history and sensitization profile).

The OFC was performed under an allergist’s supervision, and it was carried out according to international standards [18,19] adapted to the context of a one-day hospital setting [20]. The OFC was usually proposed to healthy children and postponed in case of acute diseases like fever, infectious gastroenteritis, or bronchitis. The protocol used for the nuts OFC is summarized in Table 1. The increasing doses were given every 20 min until completing the protocol or reaching the threshold dose for reaction. The OFC was considered positive if there were objective signs like urticaria, angioedema, vomiting, diarrhea, bronchospasm, hoarse voice, rhinitis, conjunctivitis, hypotension, or loss of consciousness within two hours after administration of the last food dose, which is the frame time of observation in the hospital setting for IgE-mediated food allergies. If there were reactions, patients were treated as needed and observed for a minimum of two hours until the clinical manifestations of the reaction resolved.

According to Niggeman’s classification, the OFC clinical manifestations were classified as mild, moderate, and severe [21]. Moreover, for any reaction, we described the threshold dose of the culprit nut (the maximum tolerated dose during the OFC) and its corresponding dose of nut protein. The same classification was used to define the severity of the reaction in the clinical history.

**Figure 1:**
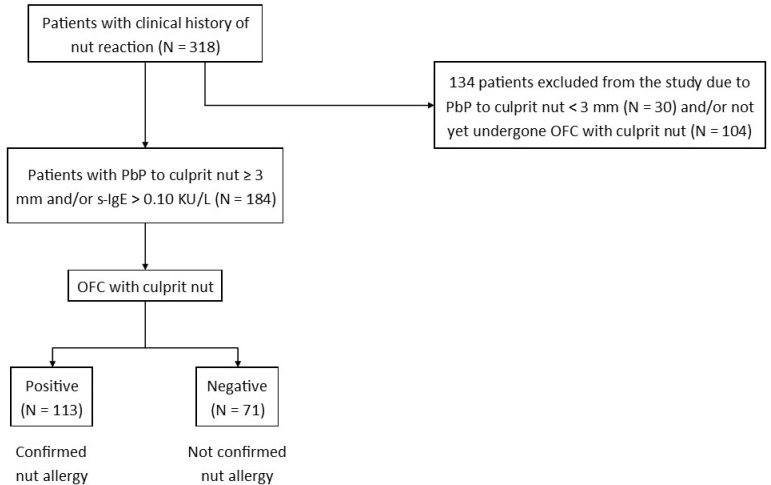
Flow chart used for the diagnostic selection of children with a confirmed nut allergy. Legend: N, number; OFC, oral food challenge; PbP, prick by prick; s-IgE, serum-specific IgE.

Finally, the studied population was represented by children with confirmed nut allergies, comprising a clinical history of nut reactions, plus positive PbP and/or s-IgE to nut plus positive nut OFC. The diagnostic selection of children with nut allergies is shown in Figure 1. Subsequently, the patients were divided into groups according to the nut responsible for the first reaction in chronological order (according to clinical history). Then, demographic characteristics (gender, age, age at first nut reaction), coexisting allergic diseases (atopic dermatitis, asthma, rhinitis, another food allergy), familiar history of allergy, values of PbP to nut and s-IgE to nut, characteristics of OFC, and clinical manifestations of the first reaction to the nut were extrapolated through chart review. Finally, these characteristics were compared between the various nuts.

Statistical analyses were performed using OpenEpi (version 3.01; Atlanta, GA, USA), Microsoft Excel (2013 version, Redmond, WA, USA), and SPSS (IBM SPSS Statistics 22, Chicago, IL, USA). Qualitative data were expressed as counts and percentages; quantitative data were expressed as mean value ± standard deviation or median value and minimum–maximum value. Data distribution was verified with the Shapiro–Wilk test and equality of variance with the Hartley F-test. Differences between continuous variables were calculated using the Student’s t-test and the one-way analysis of variance (ANOVA). Associations between categorical variables were obtained with Fisher’s exact test and chi-square test. A *p*-value of less than 0.05 was considered statistically significant.

## 3. Results

We reviewed the clinical charts of 318 children (201 males (63%) and 117 females (37%)) with a clinical history of nut reaction to the following nuts according to the first reaction in chronological order: hazelnut (112; 35%), walnut (90; 28%), peanut (58; 18%), pine nut (30; 9%), cashew (14; 4%), pistachio (8; 3%), and almond (6; 2%). No patients reported reactions to macadamia, pecan, or Brazil nuts. Overall, 184 subjects had a positive PbP and/or s-IgE to nuts and underwent OFC with the culprit nut. The OFC was negative in 71 children (39%). Conversely, 113 subjects (61%) had a positive OFC and, therefore, a nut allergy was confirmed. The result of the OFC was independent of the type of nut tested (*p* = 0.10).

The demographic characteristics of 113 children with a confirmed nut allergy are reported in Table 2. According to the nut responsible for the first reaction considered in chronological order, we identified six different groups of patients: cashew (4; 4%), hazelnut (43; 38%), peanut (22; 19%), pine nut (11; 10%), pistachio (1; 1%), and walnut (32; 28%). The demographic characteristics of patients divided for the different nuts are summarized in Table 2.

There were no statistically significant differences in gender, concurrent allergic diseases, familiar history positive for allergic diseases, or mean values of PbP and s-IgE to nuts between the various nuts (Table 2). Conversely, the difference in mean age at the first nut reaction was statistically significant (*p* = 0.00017) for the pine nut group, which was higher (8.6 ± 3.7 years) than the mean age at the first reaction to the other nuts (hazelnut 3.7 ± 3.2 years, *p* = 0.00008; peanut 5 ± 3.4 years, *p* = 0.009; walnut 4.2 ± 2.7 years, *p* = 0.0001) except cashew *(p* = 0.97). Other differences in mean age were confirmed for the cashew (8.5 ± 5.9 years) and the hazelnut groups (3.7 ± 3.2 years; *p* = 0.012) but without evidence of statistical relevance with the remaining nuts.

Seventy-nine subjects (70%) denied other allergic food reactions, concurrent or previous, based on their clinical history. Among the 34 children with food co-allergies (30%), the most frequent was egg (17; 50%), followed by milk (14; 41%), fresh fruit (13; 38%), both egg and milk (11; 32%), fish/clams (9; 26%), cereals (3; 9%), legumes (2; 6%), and seeds (1; 3%). The frequency of food co-allergies did not differ between the six nuts groups (Table 2). As regards nut co-allergies, over 75% of subjects in every group, except for the pistachio one, reported a single nut reaction (Table 3), without statistical difference between groups (*p* = 0.45).

Fifty-nine patients (52%) referenced a history of respiratory allergy (asthma and/or oculorhinitis). Grass pollen allergy was the most frequent among the pollen species tested (40; 68%), followed by cypress and birch (23; 59%).

Furthermore, 54 patients out of 113 (48%) had a history of anaphylaxis to nuts as the first reaction in chronological order: 25 patients (46% of anaphylaxis) reported moderate reactions while 19 (35% of anaphylaxis) reported severe ones. In 10 patients (19% of anaphylaxis), the severity of anaphylaxis at the first nut reaction was unknown. The occurrence of anaphylaxis (*p* = 0.16) and its severity at the first reaction in chronological order (moderate *p* = 0.77; severe *p* = 0.10) were not statistically different between the various nuts. The PbP and s-IgE values did not differ between the various nuts according to the severity of the first nut reaction. Moreover, in case of positive OFC, they did not differ among nuts according to the severity of the reaction (Table 4).

A complete molecular analysis was performed in 62 out of 97 eligible patients (64%): The highest adherence was obtained in the peanut group (86%), followed by the hazelnut (63%) and then walnut (50%) groups. Thus, the available molecular components were detected at least in 50% of the eligible population (Table 5). The mean values of molecular allergens did not correlate with the severity of the first nut reaction. Moreover, in case of positive OFC, they did not differ according to the severity of the reaction (Table 5).

**Figure 2:**
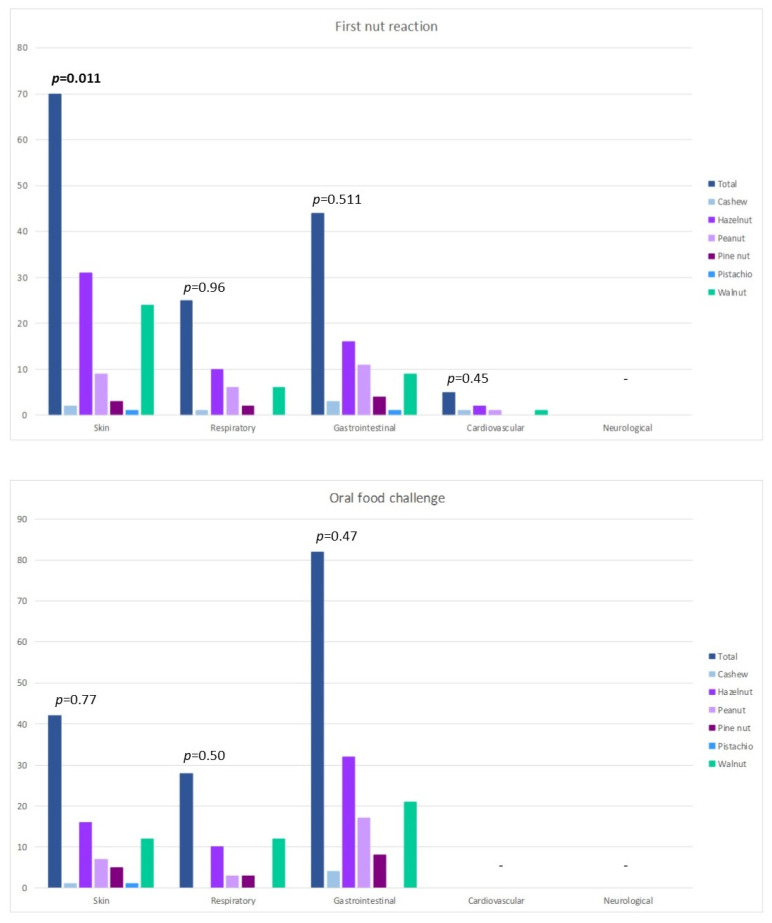
System involvement during the first nut reaction and the positive oral food challenge.

During the first nut reaction, cutaneous involvement was the most frequent reaction (70; 62%), followed by gastrointestinal (44; 39%) and then respiratory clinical manifestations (25; 22%). Seven children (6%) reported signs and symptoms by contact. No one presented neurological involvement. Instead, five children (4%) referred to cardiovascular manifestations, described only in patients with anaphylaxis. During the OFCs, the signs and symptoms involved mainly the gastrointestinal system (82; 73%), followed by the cutaneous (42; 37%) and respiratory systems (28; 25%). No one presented cardiovascular or neurological involvement. The system involvement during the first nut reaction and the OFC is reported in Figure 2. The clinical features of the reactions did not differ between the various nuts (Figure 2), except for cutaneous involvement at the first nut reaction (*p* = 0.011), referred to mainly in the hazelnut and walnut groups.

In total, 91 out of 113 subjects (81%) with clinical manifestations during OFC presented a mild reaction with single-system involvement. The remaining 22 subjects had anaphylaxis: moderate in 13 children (11%) and severe in 9 children (8%). Table 6 shows the severity of the reaction during the OFC. The severity of the reaction did not depend on the type of nut tested (Table 6).

The mean dose of nut proteins ingested was 283 mg ± 630 mg (range 2–4500 mg) in mild reactions during OFC, 376 mg ± 686 mg (range 20–2500 mg) in moderate reactions, and 69 mg ± 70 mg (range 5–200 mg) in severe ones (Table 6). The dose of nut proteins ingested was significantly lower in severe reactions if compared with the mild ones (*p* = 0.003) but not with the moderate ones (*p* = 0.13). Furthermore, there were no statistically significant differences between the severity of reaction during the OFC and the mean dose of proteins ingested between the different nuts (mild, *p* = 0.65; moderate, *p* = 0.36; mild, *p* = 0.64) (Table 6).

Furthermore, 88 out of 113 children (78%) with a positive OFC showed a spontaneous resolution of signs and symptoms. The remaining 25 subjects (22%) with reactions during the OFC required the administration of therapy: 19 (76%) were treated with oral antihistamines, 20 (80%) with oral corticosteroids, and 3 (12%) with inhaled bronchodilators. None needed the administration of injectable epinephrine. None required hospitalization or intensive care assistance. The need for therapy did not depend on the type of nut tested (*p* = 0.53).

## 4. Discussion

To our knowledge, this is the first Italian study to provide a comprehensive characterization of children with nut allergies [15], although a Turkish study has recently been published on the same topic [22]. Indeed, most of the studies about nut allergies have focused on a single type of nut, especially peanut, walnut, or hazelnut, or summarized the main characteristics of nut allergies without making a distinction between the various kinds of nuts [11,23,24,25,26]. Conversely, we retrospectively analyzed the demographic, clinical, and allergological characteristics of Italian children with nut allergies and compared them.

From our experience, in Italian children, nut allergies are more common in male subjects, and allergies to hazelnuts and walnuts were the most observed nut allergies, as stated by a previous Spanish study [27]; peanut allergies were frequently observed as well. Furthermore, most of the first nut reactions occurred between 2 and 5 years (mean age 4.7 ± 3.6 years), which is later in comparison with those in the available literature [13,22]. The underlying reason for a later age of onset of the signs and symptoms may be due to the high percentage of familial history of allergy in our population, which leads the parents to introduce the nuts in the diet later for the fear of possible allergic reaction. The mean age of the first nut reaction was statistically higher for pine nuts (8.6 ± 3.7 years) when compared with the other nuts except for cashews (8.5 ± 5.9 years). On the contrary, in the hazelnut group, the mean age of the first nut reaction (3.7 ± 3.2 years) was lower than with other nuts, even without a statistical significance. These differences may be related to the low number of patients included in the study. The majority of patients in our population had a familiar history of allergy (75%) and/or concomitant allergic disorders (68%), including asthma, atopic dermatitis, and allergic rhinitis. Among these, allergic rhinitis was the most common allergic disorder (43%), followed by atopic dermatitis (41%). The percentage of children with allergic rhinitis and a concomitant food allergy is in line with the literature (33–40%) [28].

In subjects with food co-allergies, the most frequent foods involved were egg (50%), milk (41%), and concomitant egg and milk allergies (32%), according to the literature [22]. Most of our patients (over 75% in each group) reported a single nut allergy, and the remaining subjects had at least one nut co-allergy. A coexistent nut allergy was also described in several studies [29]. Sicherer et al. reported that 34% of patients allergic to peanuts or nuts might present with multiple nut allergies [30]; however, further studies reported a large variation in the proportion of patients reacting to multiple nuts, ranging from 12% to 96.7% [31]. We found more single nut allergies because our population is younger (median age of 3.5 years) than the other ones. In particular, the studies of Sicherer et al., Maloney et al., Mc William et al., and Brough et al. found values of co-allergies and median age, respectively, as follows: 34% and 3.6 years, 34% and 6.1 years, 47.8% and 6 years, and 60.7% and 5.5 years [29,30,32,33]. The only study that showed a lower percentage of co-allergy (12%) has a younger population (1.3 years) [34]. It seems that the percentage of the co-allergy increases with the increasing age of the studied population. In our study, the most common co-allergy was hazelnut, mainly represented in the group of patients allergic to walnut. These data could depend on the high prevalence of hazelnut allergy in continental Europe, in which it represents the most frequent nut allergy [8].

Anaphylaxis as the first clinical manifestation of nut allergy occurred in over 40% of our population, similarly to the Turkish study [22]. In agreement with the literature, the most common presenting signs and symptoms at initial diagnosis of nut allergy were skin manifestations (62%), including hives, itching, flushing, and/or rash [35,36]. The cutaneous involvement was followed by gastrointestinal (39%) and respiratory (2%) ones. In the same way, the characteristic of the population (gender, familiar history of allergic diseases, concurrent allergic diseases, food and/or nut co-allergy) did not differ between the various nuts, except for the mean age of the first nut reaction, as previously mentioned. Finally, we were unable to find a statistical association between the severity of nut reaction (first one in chronological order according to clinical history and during OFC), the mean values of allergological tests (PbP, s-IgE, molecular components), and the type of nut involved.

Among positive nut OFCs, we observed 22 severe reactions (19%). However, none of these required the injection of epinephrine, hospitalization, or intensive care assistance and the clinical manifestations resolved with oral antihistamines and corticosteroids. In the literature, the occurrence of anaphylaxis during the OFC depends on the type of food tested and, for TN and peanut, it ranges from 8% to 70% according to the different studies [37,38,39]. In the remaining population (81%), clinical manifestations during the OFC involved a single system (mainly the gastrointestinal one), according to different experiences carried out on a wide range of foods [40], and resolved spontaneously, confirming the safety of the OFC in children with suspected food allergies [36,38,39]. Thus, the clinical features, the severity of the reactions during the OFC, and the need for therapy did not depend on the type of nuts tested. Conversely, the mean dose of nut proteins ingested differed according to the severity of reactions during the OFC, with a lower threshold of doses observed in severe reactions.

The limitation of this observational study is the heterogeneous number of children retrospectively recruited for each kind of nut. These data could hide real differences between the various nut groups, but they could be informative as well, strictly connected to the characteristics of children with a nut allergy referred to our center, a tertiary-care pediatric hospital. However, as this study was carried out in a single allergy unit, the reference center for the region, our results could have limited applicability to other centers and regions. Another limitation is the lack of a complete molecular analysis of patients before the OFC was performed per clinical criteria. Therefore, only 64% of the eligible population received a molecular analysis; for this reason, it was not possible to clearly discriminate patients with primary nut allergy versus PFS/OAS. Finally, due to the small number of patients in the cashew and pistachio groups, the results concerning them should be interpreted with caution.

On the other hand, the strength of this study is the clear-cut selection of the studied population with an OFC-confirmed nut allergy and the summary of the main characteristics of the nut allergy, taking into account differences between the various kind of nuts. Finally, our study also confirmed the safety of an OFC performed by experienced personnel on selected subjects.

## 5. Conclusions

In conclusion, from our experience, the majority of children with an OFC-confirmed nut allergy have a familial history of allergy (75%) and/or concomitant allergic disorders (68%). Moreover, anaphylaxis is the first manifestation of nut allergy in a high percentage of children (48%), and the presence of anaphylaxis or severe reactions as the first clinical manifestation of a nut allergy does not differ among the various nuts. The clinical and allergological characteristics of children with nut allergies described in our study are similar to other international studies: hazelnut is the most frequent nut for referral. Over three out of four subjects have a single nut allergy. Finally, during the OFCs, the signs and symptoms involved mainly the gastrointestinal system (73%), with cases resolved spontaneously in most cases and infrequent severe reactions (19%), confirming the safety of OFCs in children with suspected food allergies. These results appear important to define a comprehensive characterization of children with nut allergies in Italy. However, the results should be confirmed by extensive data from international cohorts. 

## Figures and Tables

**Table 1:** Nut oral food challenge protocol.

	Dose (mg)	Almond (mg of Protein)	Cashew (mg of Protein)	Hazelnut (mg of Protein)	Peanut (mg of Protein)	Pine Nut (mg of Protein)	Pistachio (mg of Protein)	Walnut (mg of Protein)
	5	1.05	0.9	0.7	1.3	0.7	1	0.75
	10	2.1	1.8	1.4	2.6	1.4	2	1.5
	25	5.25	4.5	3.5	6.5	3.5	5	3.75
	50	10.5	9	7	13	7	10	7.5
	100	21	18	14	26	14	20	15
	150	31.5	27	21	39	21	30	22.5
	300	63	54	42	78	42	60	45
	600	126	108	84	156	84	120	90
	1200	252	216	168	312	168	240	180
	2000	420	360	280	520	280	400	300
	4000	840	720	560	1040	560	800	600
Cumulative dose	8440	1172.4	1519.2	1181.6	2194.4	1181.6	1688	1266

**Table 2:** Demographic characteristics of patients with a nut allergy.

	Total (N = 113)	Cashew (N = 4)	Hazelnut (N = 43)	Peanut (N = 22)	Pine Nut (N = 11)	Pistachio (N = 1)	Walnut (N = 32)	*p*
Male (N = %)	74; 65	3; 75	33; 77	12; 55	5; 45	1; 100	20; 62	0.27
Age (months) (median; min; max)	42; 8; 175							
AD (N = %)	46; 41	0; 0	20; 47	6; 27	4; 36	1; 100	15; 47	0.22
Asthma (N = %)	33; 29	0; 0	14; 33	5; 23	5; 45	1; 100	8; 25	0.27
Rhinitis (N = %)	49; 43	1; 25	21; 49	7; 32	3; 27	1; 100	16; 50	0.38
Other FA (N = %)	34; 30	2; 50	17; 40	2; 9	2; 18	1; 100	10; 31	0.07
Family history of allergy (N = %)	85; 75	4; 100	33; 77	13; 59	9; 81	1; 100	25; 78	0.39
Age at first reaction (months) (mean ± SD; min; max)	57 ± 43; 8; 175	102 ± 71; 24; 172	45 ± 39; 8; 175	60 ± 41; 18; 154	103 ± 44; 48; 174	-	50 ± 33; 12; 125	0.00017
PbP (mm) (mean ± SD; min; max)	7 ± 3; 3; 15	8 ± 2; 6; 10	7 ± 3; 3; 15	6 ± 3; 3; 10	7 ± 2; 3; 10	-	7 ± 3; 3; 15	0.47
s-IgE (KU/L) (mean ± SD; min; max)	21 ± 32; 0.11; 100	3 ± 2; 1.7; 4.93	26 ± 35; 0.16; 100	31 ± 41; 0.12; 100	9 ± 19; 0.11; 66.3	-	14 ± 25; 0.3; 96.2	0.94

Legend: AD, atopic dermatitis; FA, food allergy; Max, maximum; Min, minimum; N, number; PbP, prick by prick; s-IgE, serum-specific IgE; SD, standard deviation; %, percentage.

**Table 3:** Nut co-allergy.

Nut	Other Nuts Allergies					
	Cashew (N = %)	Hazelnut (N = %)	Peanut (N = %)	Pine Nut (N = %)	Pistachio (N = %)	Walnut (N = %)
Cashew (N = 4)	-	0; 0	0; 0	0; 0	1; 25	0; 0
Hazelnut (N = 43)	2; 5		3; 7	2; 5	1; 2	3; 7
Peanut (N = 22)	0; 0	2; 9		0; 0	0; 0	1; 5
Pine nut (N = 11)	1; 9	1; 9	0; 0		0; 0	2; 18
Pistachio (N = 1)	0; 0	1; 100	0; 0	0; 0		0; 0
Walnut (N = 32)	0; 0	6; 19	1; 3	0; 0	0; 0	

Legend: N, number; %, percentage.

**Table 4:** Prick by prick and serum-specific IgE levels to the respective nuts, severity of the first nut reaction, and severity of the positive oral food challenge.

	Severity	Cashew (N = 4)		Hazelnut (N = 43)		Peanut (N = 22)		Pine Nut (N = 11)		Walnut (N = 32)		*p*	
		PbP (mm) (Mean ± SD)	s-IgE (KU/L) (Mean ± SD)	PbP (mm) (Mean ± SD)	s-IgE (KU/L) (Mean ± SD)	PbP (mm) (Mean ± SD)	s-IgE (KU/L) (Mean ± SD)	PbP (mm) (Mean ± SD)	s-IgE (KU/L) (Mean ± SD)	PbP (mm) (Mean ± SD)	s-IgE (KU/L) (Mean ± SD)	PbP	s-IgE
First reaction	Mild	-	-	7 ± 3	26 ± 35	7 ± 3	34 ± 45	7 ± 3	1 ± 1	6 ± 3	15 ± 28	1.31	0.56
	Moderate	8 ± 3	2 ± 1	9 ± 2	45 ± 47	5 ± 2	58 ± 60	8 ± 1	6 ± 5	-	-	0.20	0.27
	Severe	-	-	6 ± 3	13 ± 21	4 ± 2	26 ± 42	-	-	7 ± 4	2 ± 2	0.31	0.80
Oral food challenge	Mild	8 ± 2	3 ± 2	7 ± 3	24 ± 35	6 ± 2	29 ± 41	7 ± 2	11 ± 23	6 ± 3	8 ± 11	0.52	0.78
	Moderate	-	-	6 ± 1	45 ± 34	6 ± 4	56 ± 43	-	-	9 ± 5	54 ± 49	0.40	0.84
	Severe	-	-	6 ± 3	-	-	-	6 ± 1	3 ± 3	6 ± 3	3 ± 3	0.97	0.76

Legend: N, number; PbP, prick by prick; s-IgE, serum-specific IgE; SD, standard deviation.

**Table 5:** Molecular allergens, severity of the first nut reaction, and severity of the positive oral food challenge.

			First Reaction				Oral Food Challenge			
Molecular Allergens	Available Data (N=%)	Value (KU/L) (Mean ± SD; Min; Max)	Mild (KU/L) (Mean ± SD)	Moderate (KU/L) (Mean ± SD)	Severe (KU/L) (Mean ± SD)	*p*	Mild (KU/L) (Mean ± SD)	Moderate (KU/L) (Mean ± SD)	Severe (KU/L) (Mean ± SD)	*p*
Ara h 1	22; 100	33 ± 41; 0.15; 100	46 ± 51	28 ± 32	39 ± 40	0.80	34 ± 42	27 ± 45	-	0.98
Ara h 2	22; 100	38 ± 39; 0.6; 100	41 ± 47	32 ± 33	48 ± 42	0.92	34 ± 39	59 ± 45	-	0.64
Ara h 3	21; 95	19 ± 32; 0.11; 100	39 ± 43	11 ± 14	3 ± 3	0.88	20 ± 33	-	-	-
Ara h 8	21; 95	1 ± 1; 0.15; 2.17	1 ±1	-	1 ± 1	0.53	0 ± 0	-	-	-
Ara h 9	21; 95	16 ± 22; 0.88; 40.8	16 ± 22	-	-	-	3 ± 4	-	-	-
Jug r 1	16; 50	11 ± 22; 0.27; 88.3	4 ± 4	-	-	-	13 ± 25	4 ± 5	4 ± 4	0.93
Jur r 3	16; 50	3 ± 6; 0.12; 16	4 ± 7	-	-	-	3 ± 7	-	-	-
Cor a 1	32; 74	8 ± 11; 0.16; 32.3	8 ± 13	12 ± 14	5 ± 7	0.42	7 ± 10	-	-	-
Cor a 8	32; 74	5 ± 11; 0.11; 36.6	3 ± 4	0 ± 0	20 ± 23	0.51	6 ± 11	-	-	-
Cor a 9	33; 77	22 ± 36; 0.11; 100	21 ± 33	50 ± 57	9 ± 18	0.14	20 ± 34	49 ± 20	-	0.92
Cor a 14	31; 71	16 ± 23; 0.11; 90	19 ± 27	16 ± 17	7 ± 10	0.08	17 ± 24	12 ± 12	-	0.95

Legend: Max, maximum; Min, minimum; N, number; SD, standard deviation; %, percentage.

**Table 6:** Severity of reaction of the positive oral food challenge and dose of nut ingested.

	Total (N = 113)	Cashew (N = 4)	Hazelnut (N = 43)	Peanut (N = 22)	Pine Nut (N = 11)	Pistachio (N = 1)	Walnut (N = 32)	*p*
Mild (N = %)	91; 81	4; 100	36; 84	18; 82	8; 73	1; 100	24; 75	0.77
Protein ingested (mean (mg) ± SD)	283 ± 630	79 ± 35	279 ± 518	472 ± 1054	199 ± 350	-	221 ± 478	0.65
Moderate (N = %)	13; 11	0; 0	5; 12	3; 14	1; 9	0; 0	4; 12	0.97
Protein ingested (mean (mg) ± SD)	376 ± 686	-	764 ± 1074	123 ± 75	-	-	168 ± 47	0.36
Severe (N = %)	9; 8	0; 0	2; 5	1; 5	2; 18	0; 0	4; 12	0.58
Protein ingested (mean (mg) ± SD)	69 ± 72	-	43 ± 32	-	104 ± 136	-	44 ± 56	0.64

Legend: N, number; %, percentage.

## Data Availability

Aggregate analyses are available on reasonable request to the corresponding author.

## Abbreviations

OFC	oral food challenge
PbP	prick by prick
s-IgE	serum-specific IgE
SPT	skin prick test
TN	tree nuts
